# Clinical Lectures on Certain Diseases of the Urinary Organs; and on Dropsies

**Published:** 1857-06

**Authors:** 


### BOOK NOTICES.

*Clinical Lectures on Certain Diseases of the Urinary Organs: and on Dropsies.* By ROBERT BENTLY TODD, M.D., F.R.S., &c., &c. Published by Blanchard & Lea, Philadelphia. For sale by Keen & Lee, Chicago.

No one familiar with Dr. Todd's "*Clinical Lectures on Diseases of the Nervous System,*" need be reminded of the eminently instructive style and highly practical value, which his productions possess. Written, as they are, amid the daily scenes of hospital life and duty, by one so peculiarly adapted to the occupation of teaching, they come to us filled with the rich experience thus afforded, so moulded and shaped, and so well founded on careful and unbiased observation, that we no longer look upon the diagnosis of disease, as difficult, and, at times impossible, but, rather, as requiring only a careful analysis to completely comprehend it. Indeed, were we disposed to dissent from any part of this or his former work, it would be the regularity with which he represents the clinical phenomena attendant on different forms of disease affecting the same organ; and, the apparent ease with which he would have us arrive at difficult and delicate points in diagnoses. We are well aware that practice and experience will do much, very much, toward making us experts, but yet, with all this, we often find, in actual practice, the symptoms so various and complicated that considerable doubt will, in spite of us, remain in regard to the precise nature of the difficulty. In the preface, he says; "My aim has been to teach by examples; to inculcate cardinal points of diagnosis, treatment, and pathology by observations made at the bedside, and by illustrations drawn from suitable cases." His method is one that commends itself to the good opinion of every sensible man, and the interest and instruction derived from the perusal of the work now under consideration, interspersed as it is by illustrative cases, at once demonstrate its superior merit. The work before us contains 270 pages, divided into sixteen lectures. The first two lectures are on Hæmaturia; which he has divided for clinical purposes into *Vesical* and *Renal.* After giving the characteristic symptoms of each, and the points for a differential diagnosis, he proceeds to the detail of illustrative cases. For the purpose of indicating the peculiar characteristics of *renal* hæmaturia, he has detailed nine cases, which, for aptness and instructiveness, could not have been better selected or given in better style. He regards this form of hæmaturia as being dependent on an irritated or inflammatory condition of the kidney, and as being of two kinds: *active* and *passive.* As regards active and passive hemorrhages, he says: "In *active* hemorrhage, the rupture of the vessels arises from the attraction of an inordinate quantity of blood to them; in *passive* hemorrhage the same rupture arises not simply from the quanity of contained blood, but, rather, from its depraved quality; and the ill-nourished and weakened condition of the coats of the vessels themselves, which give way on the slightest increase of pressure. And I would especially direct your attention to the fact, that the hemorrhage of the *active* form may, by long continuance or by any cause tending to impoverish the blood and to weaken the general nutrition, degenerate into that of the *passive* kind. Vesical Hæmaturia, he considers as dependant on three causes; *first,* cystitis; *second,* retention of urine, and generation of carb ammon, by the decomposition of of urea; and *third,* the presence of a fungoid growth from the mucus membrane of the organ. Four illustrative cases are given and the subject closed by timely suggestions in regard to treatment. Lectures III. and IV. are devoted to the consideration of "*those Diseases of the Kidney with which are associated an Albuminous condition of the Urine and Dropsy.*" These cases, he says, naturally divide themselves into two great classes. "Of these, the *first* includes those cases which are distinctly and decidedly dropsical; the *second* consists of those cases in which the dropsy attracts but little attention, is very variable in amount, being in some cases considerable, but never excessive, while in others it may be very slight indeed, or even altogether absent; and in some instances, the disease may go on for a long time, and, possibly, destroy the patient's life without giving rise to the least œdema." He considers those cases belonging to the first class; namely, those which are distinctly dropsical, as being generally *acute*, and arising from cold or following scarlet fever. The second class of cases, viz., those in which the dropsy is not a prominent feature, he divides into three varieties dependent on the quantity and quality of the urine. He also regards the dropsy which accompanies these cases as being *chronic*, thus constituting the essential difference between this and the former class. These cases, when considered in reference to the general symptoms, exhibit a good deal of variety. The urine varies in amount, quality, density, and color. The albumen and dropsy, both important symptoms, are likewise subject to very great variations, and hence, we find, that he has sought to associate and reconcile all these changes, with certain pathological conditions of the kidney. Of these morbid conditions, he mentions two, which are essentially different, and with which are associated different clinical phenomena. The two morbid states of the organ are: *first*, those in which there is an *increase* of size and weight; and *second*, those in which there is a *decrease* of size and weight. Two forms of enlargement are mentioned; one, in which the pathological condition comes on *rapidly* and gives rise to *acute* symptoms; the other, in which the morbid process is *slow*, and produces symptoms of a *chronic* nature. To the first (acute), belong those cases of acute dropsy, dependant on scarlet fever and exposure; also, to phlegmonoid inflammation of the organ. To the second (chronic) belong those cases of *fatty and waxy degeneration* of the organ. To illustrate, by examples, the distinctive features of each of these classes, he has given the clinical history of three cases. *First*, a case of *fatty degeneration; second*, a case of *waxy degeneration*. Each of these cases afford an example of *chronic* enlargement, in which dropsy is not a prominent feature. The third case is given to illustrate the *chronic contracted kidney*. He has dwelt at some length on the differential diagnosis of these different forms of renal disease, and on the pathological condition which belongs to each. His remarks deserve the careful consideration of every intelligent and thinking man, but whether we shall be able to carry them to the bed side, and then say, these signs are those which belong to this or that form, with satisfaction to ourselves, and profit to our patient, can only be determined by actual trial. Theories are always convenient, and we all like to act upon them; but yet, they often require so great a stretch of the imagination as to afford very little satisfaction. Lecture V. is short, but contains some excellent remarks on the "*General Doctrines of Dropsy*," with the history of a very interesting case, showing how it may be produced by pressure on a urinous trunk, and also, how it may assist in forming a correct diagnosis; however, we may pass it over without any special comment. Lectures VI. and VII. comprise the more particular study of "*Dropsy after Scarlet Fever.*" After describing the rationale of its mode of invasion, and also the condition of the urine, he mentions *three* necessary elements for its production. These are: *first*, an irritated state of the kidney; *second*, an analogous morbid state of the skin; and *thirdly*, a certain depravity of the blood. Either of these three conditions being absent, he believes that "though we may have a threatening of dropsy, the full result will not follow." A case is given to show the ordinary clinical history of this form of dropsy, and to illustrate the existence of the three necessary elements, before mentioned. He has dwelt at length on the concurrence of this morbid state of the kidney, skin, and blood, and sought to establish a theory which would explain the occurrence of the dropsy, and the clinical phenomena which these cases present. His theory is, indeed, a plausible one; but, that he entertains a doubt that it may not stand the test of future observation, his closing remarks plainly indicate. He says, "such is my theory of the dropsy after scarlet fever. What may be the ultimate fate of it upon a larger induction of facts, I will not attempt to predict. I offer it to you now, as a convenient mode of connecting the various phenomena, which accompany, and doubtless tend to the production of, the dropsy. The treatment he recommends has for its chief object the allaying of this irritated condition of the kidney. The principal means employed for this purpose are, the hot-air bath, hydragogue cathartics, and some of the milder diuretics, which do not exercise any direct irritative action on the kidney. In addition to these means, if the kidneys do not seem to act properly, he adds local venesection over the region of the organs, late in the disease, that is, after we have succeeded in eliminating the morbid poison from the system, and only have local congestion. "This treatment is not intended to be antiphlogistic, but calmative and elimatory, soothing the renal irritation, eliminating water by the bowels, the kidneys, and the skin. The cerebral complications which not infrequently occur in this affection, are to be treated by counter-irritation; bleeding, general or local, is inadmissable. He regards this form of dropsy as being generally amenable to treatment, especially when the fever has been treated mildly, and the powers of the patient sufficiently guarded. Furthermore, contrary to the general opinion, he does not regard patients thus affected as being especially liable to subsequent renal disease. Though the affection generally terminates favorably, it does not always, and for the purpose of illustrating the prominent features of those cases most apt to prove fatal, he has detailed four cases, and given the immediate cause of death in each. In two, the fatal result depended on an œdematous condition of the lungs; one, on coma; one, on serous inflammations. There are many other points of interest in these two lectures, but as it would require a more extended notice than our space would admit to give any definite idea of their import, I shall be obliged to pass on to briefly consider the remainder of the work. Lecture VIII. "*On Acute Renal Dropsy.*" The distinction made between this, and dropsy following scarlet fever, is, that "in the latter we can trace the direct influence of an animal poison, which produces well-defined phenomena as constant and specific as those which would arise from the ingestion of arsenic, prussic acid, or strychnia. In the former, we have a dropsy of a precisely similar kind, and a state of kidney also the same; but we are unable to trace up these pathological conditions to the influence of any agent introduced from without." Illustrative cases are given, followed by general remarks, and also a detailed account of the various organic derangements which accompany and complicate this form of dropsy. He has arranged these in two classes. *First*, the *intrinsic;* and *secondly*, the *extrinsic*. The first of these derangements stand to the dropsy in the relation of cause and effect, and belong especially to the peculiar anatomical condition of the kidney, which is the same as that which occurs in dropsy after scarlet fever. He has described this morbid condition, and given an explanation of its mode of production. The *second* class of complications are, *first*, inflammations of serous membranes; *secondly*, an irritated state of the liver. The remainder of this lecture treats of the pathology, prognosis, and treatment of this disease, with more than ordinary interest. The prognosis is, on the whole, favorable: death being uncommon, unless the renal irritation proceeds so far as to produce extensive disorganization of the organ. One important element, however, in making a diagnosis, is to ascertain the previous existence of chronic renal affections; as it not infrequently happens that persons have fatty disease of the organ for a long time, without a single symptom to indicate its existence. An attack of acute renal disease, supervening on one of a chronic nature, would be much more unfavorable, and the prognosis, consequently, much graver. The principles of treatment are the same as in those cases of dropsy following scarlet fever. The eight last lectures comprise the consideration of the following subjects: Lecture IX. "on Cardiac Dropsy; X. and XI. on Ascitcs; XII. on the Gouty Kidney; XIII. and XIV. on Cases in which Pus is found in the Urine, and on Gouty Inflammation of the Bladder; XV. and XVI. on Gout. These subjects are well illustrated by cases given in admirable style, and freely commented on. In Lecture XV. "*on Gout*," he gives some very valuable suggestions in regard to treatment. A necessary feature to be borne in mind in treating this disease under any plan, is, that the great majority of cases occurring in men of good constitution will get well without any specific treatment. He supposes a hundred cases of this kind; and says, by confinement to bed, by applying warmth, by giving light, wholesome, nourishing diet, and prohibiting all kinds of liquor, seventy will get well without any unfavorable symptoms, in from four days to a fortnight. But can we not accelerate the cure? He believes we can; and recommends mild purgation, diaphoretics, warmth to the affected joint, and if the urine be acid, alkalies. He also highly recommends counter-irritation over the affected joint by means of *small* blisters. Leeching the gouty joints he condemns without hesitation; not because it does not afford marked relief, but because it is apt to leave a state of permanent weakness, from which the patient will be a long time in recovering. The treatment by Colchicum, he mentions only to condemn. He has given his reasons for abandoning the use of this drug at considerable length, and they deserve the careful consideration of his readers. His last Lecture "*on Asthmic Gout,*" affords many profitable suggestions in regard to the treatment of this form of the disorder, and especially where it attacks the internal organs, but thus barely mentioning it is all we can do in this already too long notice. The whole work is one that has been long wanted by the practitioner, as the subjects which comprise it have not, heretofore, been treated in the clinical and bedside manner pursued by Dr. Todd. We most confidently recommend it to the liberal patronage of our professional brethren. G. K. A.

